# Migraine is Related to Multiple Sclerosis Brain Lesions in the Central Pain Network with Several Migraine Phenotypes Exhibiting Different Lesion Patterns

**DOI:** 10.1007/s10548-026-01182-x

**Published:** 2026-03-04

**Authors:** Kilian Fröhlich, Kosmas Macha, Matthias Krämer, David Haupenthal, Alexander Sekita, Arnd Dörfler, Klemens Winder, Anne Mrochen

**Affiliations:** 1 Department of Neurology, University Hospital Erlangen, Schwabachanlage 6, 91054 Erlangen, Germany; 2 Department of Neuroradiology, University Hospital Erlangen, Schwabachanlage 6, 91054 Erlangen, Germany; 3 Department of Neurology, University Hospital and University of Zürich, Frauenklinikstrasse 26, Zurich, 8091 Switzerland; 4 Department of Neurology, University Hospital Giessen and Marburg, Justus-Liebig-University Giessen, Klinikstr. 33, 35392 Giessen, Germany

**Keywords:** Headache, Migraine, Multiple sclerosis, Cerebral lesions, Voxel-based lesion symptom mapping

## Abstract

Migraine is a frequent and debilitating comorbidity in multiple sclerosis (MS). Migraine headache and concomitant symptoms might be just coincidental or due to inflammatory MS activity, which is highly relevant for patients. Headache in general has been shown to be attributed to inflammatory cerebral MS lesions in the central pain matrix. The question whether migraine headache is associated with a different lesion pattern and non-painful migraine symptoms are associated with specific brain lesions sites needs further clarification. This study aimed to assess the presence of specific lesion clusters in patients with MS and comorbid migraine via voxel-based lesion symptom mapping (VLSM). Patients with multiple sclerosis and headache were prospectively identified and included in a university neurological center. As a subgroup study, patients with migraine were identified. Demographic and clinical data were assessed, and lesion volumes calculated. Cerebral lesion sites were correlated voxel-wise with presence and absence of headache using non-parametric permutation tests. A cohort of multiple sclerosis patients served as controls for the VLSM-analysis. 22 multiple sclerosis patients with migraines were included, as well as 92 controls without headache. Clinical characteristics did not differ in both groups. The VLSM-analysis showed associations between migraine and lesion clusters in the left hippocampus and bilateral thalamus. Visual aura was associated with posterior brain lesions, whilst vertigo was related to cerebellar lesions. In patients with sensory disturbances, lesions in the bilateral basal ganglia were found. MS lesions in the left hippocampus and bilateral thalamus were associated with migraine in multiple sclerosis patients. The lesion pattern indicates that migraine in MS may be facilitated by lesions in the CNS pain processing network, hypothetically through disinhibition. Visual aura in migraineurs with MS was associated with posterior, vertigo with cerebellar lesions and sensory disturbances with lesions in the basal ganglia. Hence, our data indicates that different concomitant non-painful migraine symptoms are associated with lesion sites in the related brain regions of cerebral control of the respective neurological functions. Whether MS lesions might alter brain excitability and facilitate cortical spreading depression in migraine aura remains speculative.

## Introduction

Headache is a frequent and debilitating comorbidity in Multiple Sclerosis (MS). Among various forms of headache, migraines make up the largest proportion of headache in MS.

Diagnostic interpretation of headache in MS patients can be challenging, as it sometimes may be just coincidental or due to the application of disease modifying drugs. Yet, previous studies have proposed a causal relationship, indicating that headache can sometimes be a symptom related to CNS inflammation in MS (Kister et al. 2010). 

Whether migraine or concomitant migraine symptoms are attributed to MS *is* highly relevant for patients, as they are striving for an answer for the origin of their symptoms. As it is for neurologists, as knowledge about whether headache is due to MS activity or not may potentially hold diagnostic and therapeutic implications, e.g. to pursue MR imaging or to consider immunomodulatory treatment.

Migraines have mainly been associated with lesions in the brainstem before (Tortorella et al. 2006, Marciszewski et al. 2018, Leandri et al. 1999, Gee et al. 2005, Gentile et al. 2007, Mantia 2009). Especially, new onset of migraine has been observed in patients with lesions in the trigeminal root entry zone, trigeminal nuclei and periaqueductal gray matter (Tortorella et al. 2006, Marciszewski et al. 2018, Gee et al. 2005). The trigeminocervical complex consist of major relay neurons for nociceptive afferent input from the meninges and cervical structures relevant in the development of headache. The periaqueductal gray is an important structure for pain modulation (Gee et al. 2005, Mantia 2009). 

Recent work has also revealed an association of other, especially supratentorial cerebral lesions in the central pain matrix (namely the insula, thalamus, hippocampus) to be associated with headache in MS (Frohlich et al. 2025). Moreover, other pain syndromes and autonomic symptoms like trigeminal neuralgia, bowel dysfunction and urinary incontinence have shown to be related to damage of cerebral pathways due to MS lesions (Charil et al. 2003). 

Existing studies in migraine patients consist only of case series or a visual region-of-interest-based descriptive analysis with a focus on infratentorial brain regions (Gee et al. 2005, Gentile et al. 2007). Systematic data and especially neuroimaging evidence about the association between inflammatory MS lesions and migraine in MS is still scarce (Mrabet et al. 2022, Zhang et al. 2023, Horton et al. 2023). 

To our knowledge, the association between migraine, migraine symptoms and MS lesion location in the brain has not been further examined yet.

Migraine is known for its huge variety of symptoms and phenotypes, including neurologic deficits like aura or autonomic dysfunction. As headache in general is associated with damage to several regions known to be important for central pain regulation in MS, we postulated that migraines and especially its non-painful concomitant symptoms and phenomena may be attributed to lesion patterns in distinct brain regions, too.

To overcome the mentioned disadvantages of previous studies, we therefore performed a statistical voxel-based lesion mapping analysis of the whole brain (Frohlich et al. 2018). Statistical imaging analysis like the voxel-wise lesion symptom mapping (VLSM) allows investigating voxel-by-voxel associations between cerebral lesion location and an outcome without having any a priori hypothesis (Frohlich et al. 2018, Huang et al. 2023, Bates et al. 2003, Rorden et al. 2007). 

## Methods

The aim of this lesion mapping study was to identify brain regions, which are related to the development of migraine in multiple sclerosis. We therefore performed a subgroup analysis of migraine patients out of a larger cohort of patients with headache and comorbid multiple sclerosis.

### Patients

The prospective study was approved by the local ethics committee of the Friedrich-Alexander University Erlangen-Nuremberg (No. 93_17 B).

For the study, MS patients admitted to the Department of Neurology at the University Hospital Erlangen were prospectively screened for headache between 2017 and 2023 and included, if suitable.

*Only patients with migraine onset after the first manifestation of MS were included.* Patients were excluded with a history of other cerebral conditions, or the headache was interpretated as a side effect of the treatment with disease-modifying MS drugs. Only patients with persisting headache at inclusion were considered.

Diagnosis of MS followed the recent guidelines (Polman et al. 2011, Thompson et al. 2018). 

Headache characteristics were evaluated via a written questionnaire adhering to the International Classification of Headache Disorders 2013). Other clinical parameters and examination results were derived from the written records.

As a control group, a cohort of MS patients without headache was established. All data were entered in a prospective database.

### Cerebral Imaging

All patients underwent magnetic resonance imaging (MRI) (3 Tesla, Magnetom Trio or 1.5 Tesla Siemens Magnetom Sonata, Siemens Healthcare, Erlangen, Germany) of the brain.

### VLSM

Two experienced investigators (K.F. and KW.) delineated the boundaries of the hyperintense flair lesions on anonymized imaging scans using MRIcron (www.mrico.com) (Rorden et al. 2007). Both raters were blinded to clinical parameters during imaging analysis. The MRI scan and the lesion shape were transferred into stereotaxic space using the normalization algorithm of SPM12 (http://www.fil.ion.ucl.ac.uk/spm/) and the Clinical Toolbox for SPM (http://www.mricro.com/ clinical- toolbox/ spm8- scripts). Using the MR-segment-normalize algorithm of the Clinical Toolbox, the MR images were transformed to the T1 template (Rorden et al. 2012). Lesion volumes in voxels were calculated using the non-parametric mapping (NPM) algorithm included in MRIcron. In a VLSM analysis, the lesion site was correlated with the occurrence of migraine using non-parametric permutation testing (Bates et al. 2003). All lesioned voxels were included in the analysis. A false discovery rate (FDR) correction of 0.05 was applied. The peak coordinates of the involved regions are presented in Montreal Neurological Institute (MNI)-space. In the same way, a VLSM analysis using non-parametric permutation testing was performed with subgroups of patients with migraine and MS.

### Statistical Analysis

For data analysis, a commercially available statistic program (SPSS 20.0; IBM, Armonk, NY) was used. Distribution of data was tested using Shapiro–Wilk test. Data are presented as mean and standard deviation (SD) or median and interquartile range (IQR). Normally distributed patient and control data were compared using the t-test for unpaired samples. Non-normally distributed data were compared using the Mann–Whitney U-test. Significance was assumed for *P* < 0.05.

## Results

### Patient Characteristics

48 of the identified MS patients with headaches agreed to participate in the study were included, of whom 22 patients were migraine patients and eligible for the subgroup study. 4 patients were excluded because headaches were interpretated as a side effect of the disease modifying MS medication. No patients were excluded because of comorbid other cerebral diseases. 92 MS patients were recruited as controls.

Clinical characteristics of the study participants are described in Table 1. Of the 22 patients with migraine, 20 (91%) were female and 2 (9%) were male. Among controls, 66 (72%) were female and 26 (28%) were male.

**Table 1:** Clinical and imaging characteristics of the 22 individuals with migraine headache and the 92 controls without headache in patients with multiple sclerosis

	Migraine, n =22	No headache, n = 92
Age, mean ± SD; y	40 ± 10	42 ± 11
M/F	2/20	26/66
EDSS; median, IQR	2 (1.5-3)	2 (1.5–4.5)
Disease duration, median, IQR,	69 (20–120)	36 (12–123)
MS type (PPMS/RRMS/SPMS)	0/21/1	3/66/22
Lesion volume (voxels), median, IQR	18,964 (6236–42242)	16,787 (9422–37365)

y, years; EDSS; IQR, interquartile range; SD, standard deviation; M/F, man/female ratio.

*Indicates significant difference using Mann Whitney U Test.

Of the 22 patients with migraine, 10 reported vertigos or dizziness as a concomitant migraine symptom. 7 complained about visual aura, 5 about sensory disturbances and 2 about temporary limb weakness or allodynia.

Disease duration was higher among migraineurs compared to controls; however, these results did not reach statistical significance. Other parameters did not differ between both groups.

### VLSM

**Fig. 1:**
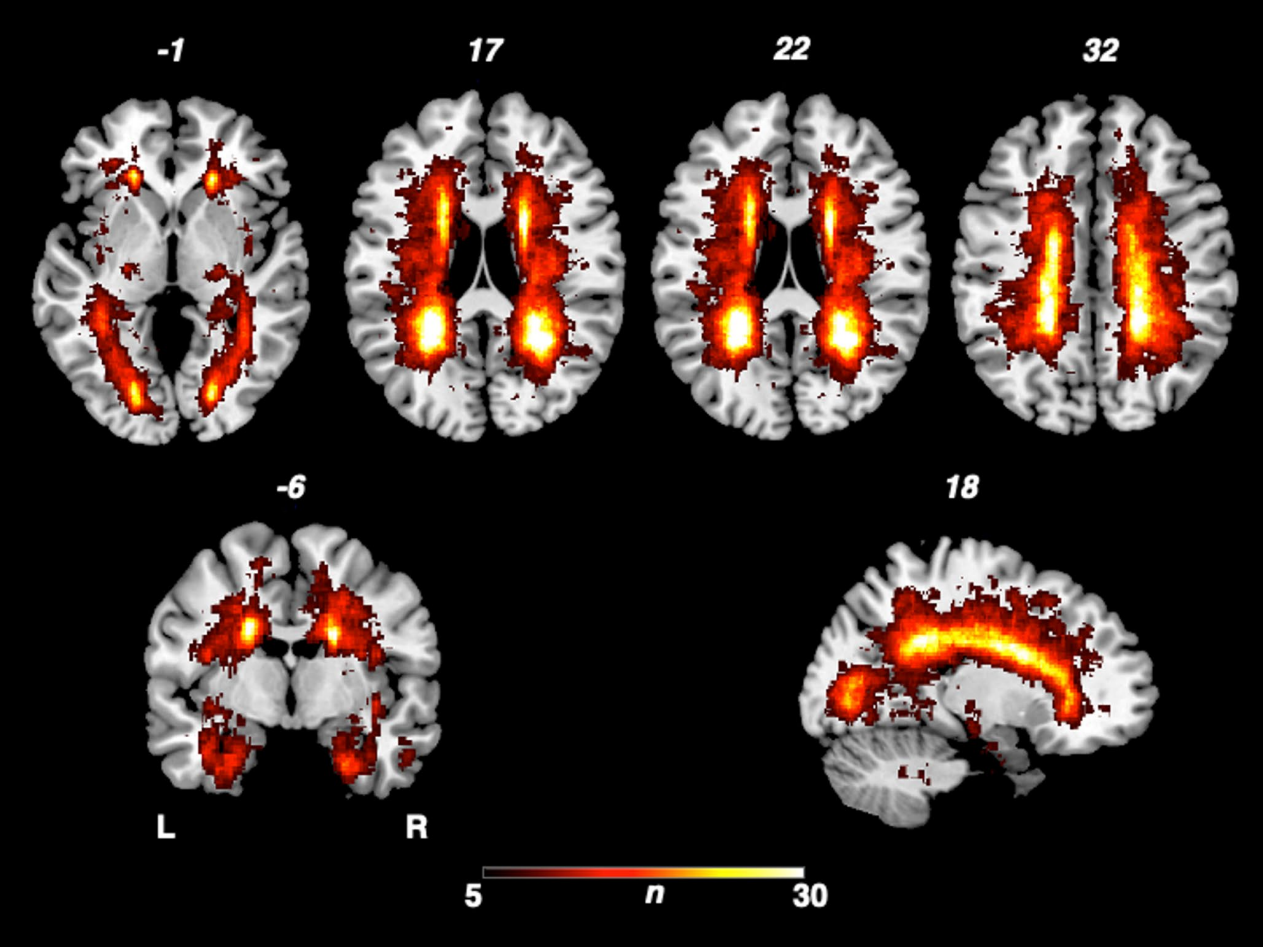
Overlap and distribution of T2 lesions of all patients. The number of overlapping lesions is illustrated by color coding. The pattern shows the highest overlay in subcortical and periventricular regions, congruent with the multiple sclerosis cohort L: left; n: number of individuals with a lesion in a given voxel; R: right

In Fig. 1, the overlap of cerebral lesions of all 114 MS patients is demonstrated, yielding similar results with the overlap of our previous study (Frohlich et al. 2025). The analysis derived a typical lesion pattern of inflammatory MS lesions with subcortical preponderance, which is illustrated in axial, sagittal and coronar view.

**Fig. 2:**
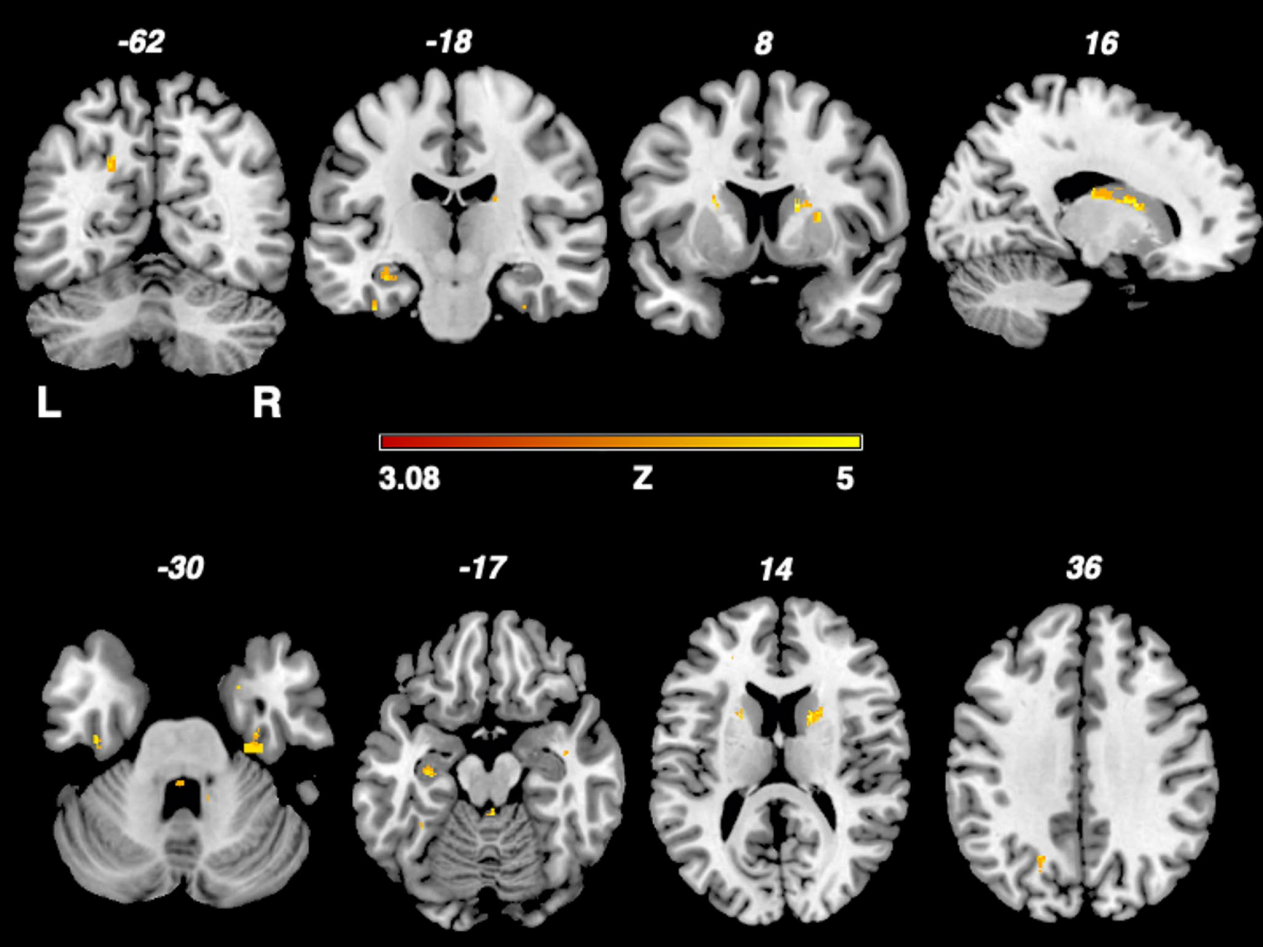
Depiction of the nonparametric Liebermeister test results. Associations of lesioned voxels with headache were found in the left insula, left hippocampus and right thalamus. Only voxels that were damaged in at least two patients were included in the Liebermeister test analysis. A family wise error (FWE) correction of *p* < 0.05 was applied. L: left; z: z-score; R: right

Figure 2 shows the results of the nonparametric Liebermeister analysis with bilateral lesion clusters in the thalamus and hippocampus, examining the 22 patients with migraine in MS, compared to 92 controls without headache.

**Fig. 3:**
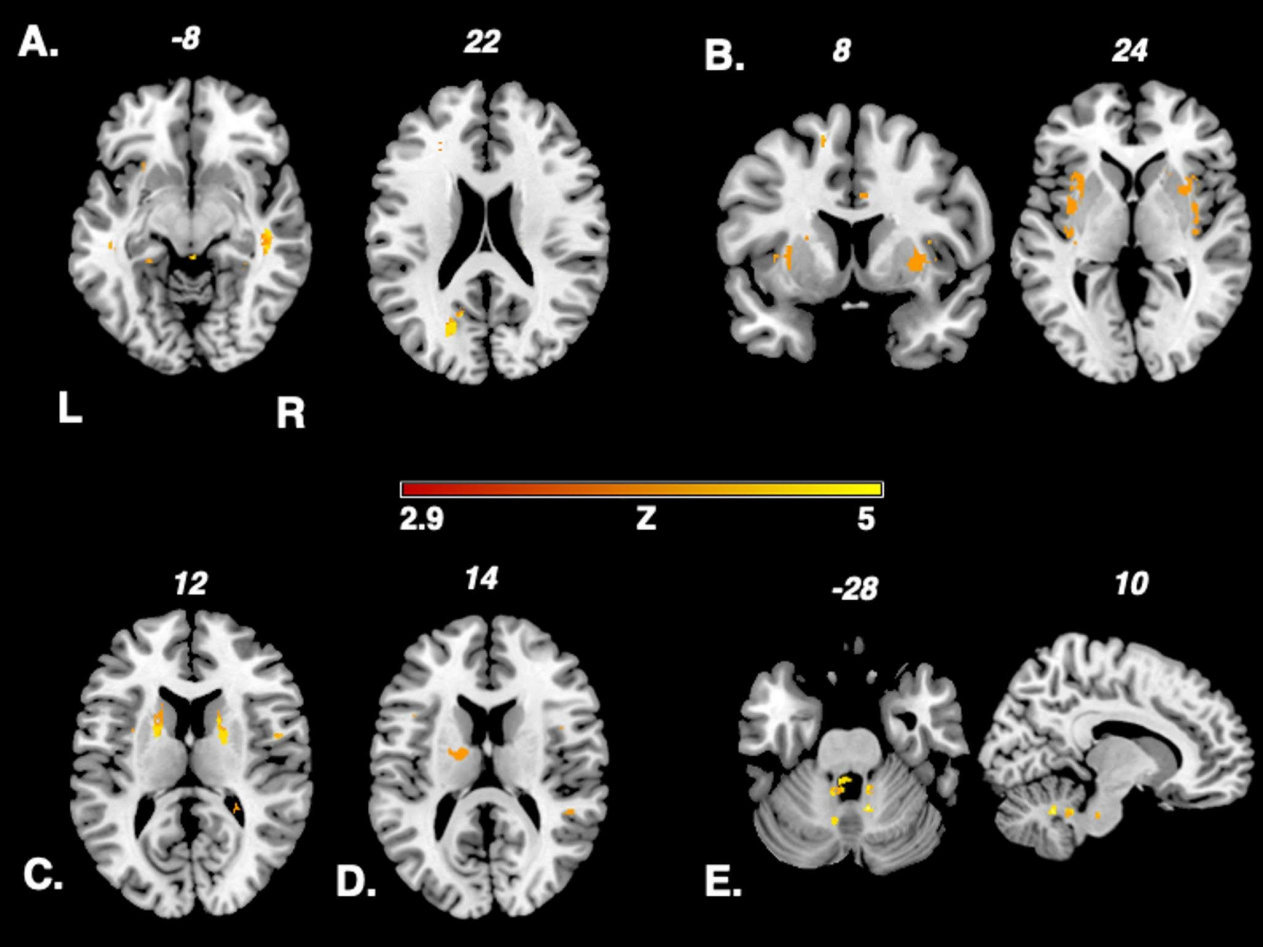
Presentation of the nonparametric Liebermeistertest results in various migraine phenotype subgroups. A: patients with visual disturbances B:aura with abnormal sensation, C: aura with hemiparesis, D: cutaneous allodynia, E: vertigo Onlyvoxels that were damaged in at least two patients were included in the Liebermeister testanalysis. A family wise error (FWE) correction of *p* < 0.05 was applied. L: left; z: z-score; R: right

Figure 3 highlights the results in the different subgroups of migraine phenotypes (aura or concomitant symptom) in the Liebermeister analysis (A: patients with visual disturbances B: aura with abnormal sensation, C: aura with hemiparesis, D: cutaneous allodynia, E: vertigo). Adding lesion volume as a regressor, the Liebermeister test remained significant, yet almost no voxels survived the analysis. Adding sex and diseased duration as covariates, the test yielded similar results to the primary analysis. We chose to present the Liebermeister test results without covariate analysis as it provided more clear results.

## Discussion

Headache in MS has previously shown to be associated with MS lesions in certain brain regions of the central pain control (Frohlich et al. 2025). The relationship between MS lesion location and migraine as well as different migraine symptoms in MS patients has not been elucidated yet. In the present study, our aim was therefore to investigate whether migraine headache and certain MS features like aura symptoms are also attributed to certain lesion patterns in the brain.

First, our study revealed a lesion cluster of bilateral lesions in the thalamus and the hippocampus to be associated with migraine in MS patients (Fig. 2). All these structures are known to be important elements of the central pain network, processing afferent pain signals. Lesions of those central nervous system structures that are involved in pain processing are known to potentially lead to alterations of pain sensation or centrally mediated pain (Frohlich et al. 2018, Sprenger et al. 2012, Seifert et al. 2016, Craig et al. 2000). 

Altogether, painful stimuli are conveyed to the periaqueductal gray matter (PAG) in the brainstem and relayed to the thalamus (Craig et al. 2000). Subsequently, nociceptive signals are projected to the insula and somatosensory cortex (Craig et al. 2000). 

As the thalamus exerts inhibitory effects via a neural pathway on the insula and the PAG (Sprenger et al. 2012), we previously proposed that a disinhibition of the endogenous pain modulation by localized thalamic lesions may facilitate the development of headache like migraine (Frohlich et al. 2025). 

The hippocampus is part of the central pain matrix and linked to other pain processing regions via pathways, namely the insula (Bornhövd et al. 2002, Ploner et al. 2010). It plays an important role in pain inhibition and modulation, especially involving the affective system and cognition (Neugebauer and Kiritoshi , Tian et al. 2016, Kong et al. 2008). 

In addition, affection of the hippocampus may lead to hyperalgesia (Tian et al. 2016, Kong et al. 2008). Hence, we hypothesize that lesions in the hippocampus may promote migraine by disinhibition of central inhibitory pain mechanisms (Craig et al. 2000). 

Taken together, our data supports the hypothesis that, in at least a proportion of headache patients with MS, strategic inflammatory lesions in distinct brain regions may play a relevant role. As the hippocampus and the thalamus are important structures in central pain regulation, the existence of lesions in these areas suggests that disinhibition resulting from inflammatory lesions may play a major role in patients with migraine an MS.

Yet, as one major finding, the analysis did not reveal a major specific and different lesion pattern of migraine headache in general, compared to patients with non-specific headache, in multiple sclerosis patients (Frohlich et al. 2025). Only an additional lesion cluster of the left thalamus was found in patients with migraine compared to patients with other types like tension-type headache in MS – a finding with unclear significance.

Second, as migraine is very heterogenous with different concomitant symptoms and aura, we therefore performed a subgroup analysis of patients with migraine in MS. Our study revealed different phenotypes and migraine symptoms to be associated with MS lesions in specific brain regions. Hypothetically, in our view, brain lesions may alter brain excitability and facilitate migraine symptoms and aura.

Various neuroimaging findings in patients with migraine with aura but without MS, yet not consistent and concluding, have been described (Karsan et al. 2023, Petrušić and Hadjikhani 2025, Zhang et al. 2023). Migraine with aura seems to be mediated by widespread brain dysfunction ictally and interictally in areas involving, but not limited to the visual cortex, other cortical areas and the thalamus (Karsan et al. 2023, Petrušić and Hadjikhani 2025, Zhang et al. 2023). 

Visual phenomena accompanying migraine like visual aura were associated with lesions in the subcortex and posterior white matter, a brain region containing the optic tract (Fig. 3, A). These findings strongly suggest that visual aura in migraine may be triggered or is facilitated through MS lesions affecting the neurons of the visual system. Consistent with our results, typical visual aura consists of scotoma or fortification figures related to posterior brain regions of the visual cortex and optic radiation within the occipital lobe (Eren et al. 2021). Furthermore, cortical spreading depression as pathophysiological correlate of visual aura has been verified in the named brain regions via neuroimaging studies (Eren et al. 2021). 

Abnormal body sensation like hypesthesia as aura symptom was related to lesions in the claustrum and adjacent to the insula (Fig. 3, B). The insula is a highly complex brain structure that, among others, plays a crucial role in processing exteroceptive as well as interoceptive sensory input (Zhang et al. 2024). Exteroceptive information, that is i.e. somatosensory signals from the outside world, are directed towards topographical organized insular subregions, processed and conveyed to the sensory cortex (Zhang et al. 2024). Following the findings of our analysis and regarding the spatial organization of the insula, we postulate that insular lesions could make patients more prone to sensory aura in migraineurs with MS.

In the 2 patients with self-reported motor weakness as aura symptom, bilateral lesions in the internal capsule were found to be associated with migraine in MS (Fig. 3, C). The significance of the lesion cluster found remains unclear, especially due to low statistical power.

A lesion cluster in proximity to the right thalamus was associated with cutaneous allodynia (Fig. 3, D). The thalamus is known, among various other functions, to serve as a major relay region for afferent sensory signals (Bernstein and Burstein 2012). For pain perception, the thalamus is believed to modulate nociceptive information before transmitting nociceptive inputs to cortical structures (Bernstein and Burstein 2012, Wang et al. 2015). Cutaneous allodynia is found in up to 60% of migraineurs and describes the painful perception innocuous stimuli of the skin (Wang et al. 2015). Central sensitization, that is lowered activation threshold of trigeminal neurons with increased responsiveness to afferent inputs has been hypothesized as major pathophysiologic mechanism in cutaneous allodynia in migraine (Wang et al. 2015). Central sensitization of third order trigeminal neurons in the posterior thalamic nuclei mediates cutaneous allodynia in migraine (Bernstein and Burstein 2012, Wang et al. 2015). We speculate that hypothetically the lesion cluster found in the thalamus may promote cutaneous allodynia, lowering the gate-keeper function of the thalamus for afferent sensory input via thalamic lesions.

In patients with vertigo or dizziness as a concomitant migraine symptom, we detected an infratentorial lesion cluster in the cerebellum (Fig. 3, E). Cerebellar lesions are known to frequently cause vertigo in various conditions like MS but also stroke or neoplasia by lesions of the vestibulocerebellar, vestibulospinal or cerebellar ocular motor system (Feil et al. 2019, Grimaldi and Manto 2012). As main mechanisms in clinical routine, cerebellar ocular motor disturbances or vestibular hyperactivity are reported (Feil et al. 2019). Vestibular hyperreactivity in cerebellar syndromes has been discussed as being caused by a dysfunction of olivocerebellar projections (Thurston et al. 1987). 

Finally, it is important to mention that other sorts of aura (e.g. dysphasia) did not show associations between MS lesions in migraineurs. Whether statistical power was too low in some subgroups or some brain regions tend to be more prone to develop aura when lesioned, remains speculative.

## Limitations

Although reported headaches were an exclusion criterion, we cannot completely exclude that headaches were also present in the control group. Due to the heterogeneity of headache and the fact that diagnosis is based on clinical features, we also cannot exclude that in a small proportion of patients, headaches may be due to the disease modifying MS medication in the study cohort, although this was an important exclusion criterion.

The number of migraineurs and statistical power is, partially, rather low for subgroup analysis. However, as VLSM is a very robust method and a larger control group was chosen, it seems unlikely that the statistical analysis may have produced false-positive results, yet more likely false-negative results.

## Conclusion

MS lesions in the bilateral thalamus and hippocampus were associated with migraine in MS patients. The lesion pattern indicates that migraine in MS may be facilitated by lesions in the CNS pain processing network, hypothetically through disinhibition. Yet, the pattern in migraine did only slightly differ from other sorts of headache or non-specific headache in MS.

Visual phenomena in migraineurs with MS were associated with posterior, vertigo with cerebellar lesions and sensory disturbances with lesions in the basal ganglia. Hence, our data indicates that different concomitant non-painful migraine symptoms are associated with lesion sites in the related brain regions of cerebral control of the respective neurological functions. Whether MS lesions might alter brain excitability and facilitate cortical spreading depression in migraine aura remains speculative.

## Data Availability

On reasonable request from the corresponding author.

## Abbreviations

MRI: Magnetic resonance imaging
VLSM: Voxel-based lesion symptom mapping
